# miR-31 is distinctively overexpressed in primary male extramammary Paget's disease

**DOI:** 10.18632/oncotarget.8230

**Published:** 2016-03-21

**Authors:** Hao Guo, Rui-Qun Qi, Ya-Ni Lv, He-Xiao Wang, Yu-Xiao Hong, Song Zheng, Jiu-Hong Li, Xing-Hua Gao, Hong-Duo Chen

**Affiliations:** ^1^ Department of Dermatology, No.1 Hospital of China Medical University, Shenyang, China

**Keywords:** miRNA, miR-31, non-melanoma skin cancer, extramammary Paget's disease, apocrine glands

## Abstract

MicroRNAs (miRNAs) are small noncoding RNAs involved in cancer development. Extramammary Paget's disease (EMPD) is a rare cutaneous malignancy and the role of miRNAs in EMPD remains unknown. Here, we used TaqMan miRNA arrays to characterize miRNA expression profile in EMPD and further validated the candidates by single RT-PCR. Total 12 cases EMPD were involved in this study. Using laser capture micro-dissection technique, we collected EMPD tumor cells (ET, *n*=12), normal epidermal cells (NE, *n*=12) and normal apocrine glands cells (NA, *n*=7). MiRNA arrays from two pairs of ET and corresponding NE showed that miR-375, miR-10b, miR-31, miR-31* were differentially expressed. The single real-time PCR (RT-PCR) further confirmed that miR-375, miR-31 and miR-31* were upregulated in EMPD cells than those of the normal epidermis and apocrine glands. Our preliminary study suggested that these miRNAs could be involved in EMPD development and miR-31 may serve as potential biomarkers of EMPD.

## INTRODUCTION

Extramammary Paget's disease (EMPD) is a cutaneous malignancy that always affects apocrine-rich areas such as the vulva, penis, scrotum and perianal area. The pathogenesis of EMPD is still unclear. It shows differentiation to an apocrine gland [1] and the bulk of evidence points to a histogenetic origin of Paget cells from apocrine glands [1–8]. MicroRNAs (miRNAs) are a class of 19-25 nucleotide noncoding RNAs that regulate diverse cellular processes, such as proliferation, differentiation, and cell death. Different miRNAs have been demonstrated to be differentially expressed in tumor tissues and contribute to cancer development [9]. There are currently no any published studies in the role miRNAs in EMPD. Here, we hypothesized that EMPD tumor cells may express a specific miRNA profile, which could contribute to EMPD development.

## RESULTS

**Figure 1:**
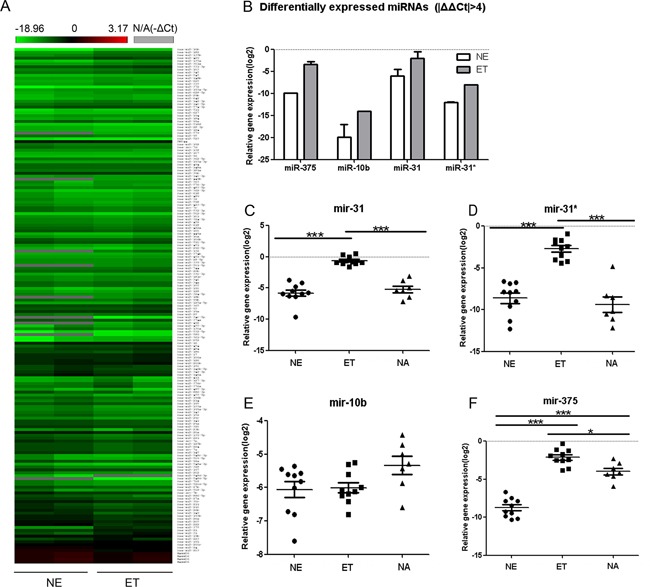
Differentially expressed miRNAs **A.** MiRNA expression profiles. MiRNA expressions two pairs of normal epidermal cells (NE) and EMPD tumor cells (ET) from same patients were profiled using TaqMan Human MicroRNA array card A and B (v2.1). The cycle threshold (Ct) values were obtained with SDS 2.3 and RQ manager 1.2 software (Applied Biosystem) and analyzed with RealTime StatMiner® 4.2 software (Integromics, Inc.). The -ΔCt [−(Ct-Ct_RNU48_)] was calculated and heat map analysis was performed with hierarchical clustering. A green-red color scale (−18.96 to 3.17) depicts normalized miRNAs expression level based on internal control (RNU48), and grey represents undetected. **B.** Different expression of miRNAsin ET and NE, based on the array analysis. The expression levels (−ΔCt) of miR-375, miR-10b, miR-31 and miR-31* in ET were much higher than that in NE. |ΔΔCt|≥4, ΔΔCt=ΔCt_ET_-ΔCt_NE_. **C–F.** The relative gene expression level (−ΔCt) of miR-31(c), miR-31*(d), miR-10b(e) and miR-375(f) in ET(n=10), NE(n=10) and normal apocrine glands cells (NA, n=7). (*:P<0.05, ***: P<0.0001).

To test our hypothesis, fresh frozen tissue samples from 12 EMPD patients were separated into EMPD tumor cells (ET, *n*=12), normal epidermal cells (NE, *n*=12) and normal apocrine glands cells (NA, *n*=7) precisely via laser capture micro-dissection. By miRNA array analysis, we got specific miRNA expression profiles using two paired samples of ET and NE. Array test was not applicable to NA due to the low quantity in each individual samples. We setup a cut off Ct value at 35. In the overall cohort, 406 miRNAs were detected, in which 253 were from pool A and 153 from pool B. Heat-map analysis was performed using the -ΔCt values (Figure 1A) which showed the consistent expression of a majority of miRNAs across different samples. Of 256 miRNAs detected in all samples, we found that four most changed miRNAs, miR-375, miR-10b, miR-31 (miR-31-5p) and miR-31* (the complementary miRNA of miR-31, miR-31-3p) were differentially expressed (|ΔΔCt|>4) between the NE and ET (Figure 1B).

The differential expression between NE and ET in miRNA array could be influenced by at least two factors: differences between normal and malignant tissues as well as the differences between the cells origin/cell types. To further validate the differentially expressed miRNA candidates mentioned above, we preformed TaqMan signal real-time PCR (RT-PCR) on miR-375, miR-10b, miR-31 and miR-31*, using the remaining samples (10 ET, 10 NE and 7 NA). (Figure 1C–1F)

Bonferroni's Multiple Comparison Test was carried out among three groups. Expression levels of miR-31 (Figure 1C) and miR-31* (Figure 1D) in ET were significantly higher than those from NE and NA (P<0.0001), while no significant differences between NE and NA (P>0.05). The expression level of miR-375 (Figure 1F) showed significant differences among the three groups, ET had the highest level of expression. The expression level of miR-10b (Figure 1E) showed no significant differences among these three groups.

## DISCUSSION

MiR-31 is expressed widely in different cell types [10–13] and has been extensively studied in different cancers. Its expression has been found to be down-regulated in human carcinomas of the prostate [14], ovary [15], and stomach [16]. Paradoxically, up-regulation of miR-31 has been shown in human cervical [17], colorectal [18, 19], liver [20] and head-and-neck squamous cell carcinomas [21, 22]. Liu et al [21] showed that miR-31 has the capacity to promote primary tumor growth in head-and- neck squamous cell carcinoma. In relation to its up-regulated expression in EMPD, we suspect that miR-31 may correlate with the development of EMPD, considering that the possible source cells from apocrine glands had moderate expression. According to the data of Mirbase, miR-31 has 176 validated target genes. Among them only SP1 (specific protein1) and AR (androgen receptor) have been studied in EMPD. While both SP1 and AR were higher expressed in EMPD through immunohistochemical stain [23–25], indicating they were unlikely to be related with the overexpression of miR-31 directly. MiR-31* is the complementary miRNA of miR-31. MiR-31*expression was consistent with miR-31 (Figure 1C, 1D), indicating a generalized deregulation in the precursor transcript from which these mature miRNAs are derived.

MiR-375 has been found to be dysregulated in numerous types of malignancy acting as either tumor suppressors or oncogenes [26–33]. We suspect the overexpression of mir-375 may correlate with the development of EMPD. In our observation, the inconsistency of miR-10b between array and single RT-PCR might be induced by the limited number of samples in the array.

This is the first report to indicate that miR-31, miR-31* and miR-375 were overexpressed in EMPD and miR-31 could be served as a potential biomarkers of EMPD. However, further studies using a large cohort are needed to identify their targets and related singling pathways involve in EMPD development.

## PATIENTS AND METHODS

### Patients

We collected archived 12 male patients (Supplementary Table S1, available online) with clinically and histologically confirmed EMPD aged from 56 to 79 year-old. These patients all came from Northeast China, and other malignancies had been ruled out via comprehensive examinations (including chest CT, abdominal & urinary system B ultrasonic and enteroscope) prior to surgery. To minimize the heterogeneity among our cancer cases, samples from perianal area were excluded in this study.

### Laser capture micro-dissection

Twelve fresh frozen EMPD tumor tissues underwent sectioning and staining. By laser capture micro-dissection (MMI cellcut), EMPD tumor cells (ET), normal epidermal cells (NE) and normal apocrine gland cells (NA) were precisely isolated, respectively (Supplementary Figure S1, available online)

### RNA isolation

All the collected tissue of these three groups underwent lysis and extraction of total RNA including miRNA by Qiagen miRNeasy Micro Kit with the protocol provided by Qiagen.

### TaqMan low-density array miRNA qRT-PCR

Due to limited RNA could be obtained using microdissection technique, a preamplification step was added per manufacture's protocol when miRNA array was performed in two paired samples of ET and NE from same patients for gross screening. The RNA was reverse transcribed using the TaqMan MiRNA Reverse Transcription Kit and the TaqMan miRNA Multiplex RT Assays, Human pool A, B (V2.1, V3.0, respectively). The expression was profiled with TaqMan Human microRNA arrays (V2.1 for pool A and V3.0 for pool B), using the manufacturer's recommended protocol (Applied Biosystems, Foster City, CA, USA).

### Single real-time PCR (RT-PCR)

Upon obtaining significantly differentially expressed miRNAs, RT-PCR was performed on specific miRNAs among the three groups of cells using the remaining samples (10 ET, 10 NE as well as 7 NA). Due to their scarcity, NA from EMPD patients could not be found in every samples.

### Data analysis

For miRNAs that were observed in all samples, cycle threshold (Ct) values were obtained with SDS 2.3 and RQ manager 1.2 software (Applied Biosystem), and ΔCt values were used in the statistical analysis. Differential expression of miRNAs were calculated with StatMiner® 4.2 (Integromics® Inc., Philadelphia, PA). -ΔCt [−(Ct-Ct_RNU48_)] was calculated and heat map analysis was performed with hierarchical clustering. Histograph and scattergraph was performed by GraphPad Prism.

Detailed methods and data analysis are shown in Supplementary Methods.

## SUPPLEMENTARY FIGURES AND TABLES




