# Supplementary material for: miR-31 is distinctively overexpressed in primary male extramammary Paget's disease
Source: Oncotarget. 2016 Mar 21;7(17):24559–63. doi: 10.18632/oncotarget.8230 (PMC5029722; doi:10.18632/oncotarget.8230)
Supplement: Supplementary file 1 [file oncotarget-07-24559-s001.pdf]

SUPPLEMENTARY METHODS

Precise cell selection via laser capture micro-dissection

We collected 12 cases of extramammary Paget's disease (EMPD) tissue with the patients permission. Fresh frozen samples underwent sectioning and staining. Laser capture micro-dissection (MMI cellcut) helped divide the samples into one of three groups precisely. The EMPD tumor cells group (ET) was composed mostly of extramammary Paget's cells along with the adherent small numbers of epidermal cells, the normal epidermal cells group (NE) was primarily keratinocytes, and the normal apocrine glands cells group (NA) is composed of normal apocrine gland cells (supplementary Figure 1). The NE and NA were collected from corresponding patient's surgical margins histologically free of tumor cells to minimize heterogeneity. About 1mg tissue of NE, 1 mg ET group and 0.3mg of NA was collected via micro-dissection from each sample (as the dosage of apocrine glands in not sufficient to collect upto 1mg and it is observed only in 7 cases).

RNA isolation

All the collected tissue of these three groups underwent lysis and extraction of total RNA including miRNA by Qiagen miRNeasy Micro Kit with the protocol provided by Qiagen. Briefly, collected tissue lysed with 750ul QIAzol lysis reagent, vortexed for 2 min, hold at room temperature for 5 minutes, add 140ul chloroform and shake vigorously for 30s, stay at room temperature for 2 min, centrifuge for 15min at 12000xg at 4°C, transfer the upper aqueous phase to a new collection tube, add 1.5 volumes of 100% ethanol and mix thoroughly. Combined the separated mixtures from the same sample and run through the same RNeasy Micro spin column. The remaining Qiagen protocol was then followed. 15ul RNase free water was used to elute in the last step. All the samples were vacuumed down to about 5ul for use. RNA quantity and quality were assessed with the Thermo Scientific Nanodrop 2000 (Thermo Fisher Scientific, Inc.).

TaqMan low-density array miRNA qRT-PCR and real-time PCR (RT-PCR)

The RNA was reverse transcribed using the TaqMan MiRNA Reverse Transcription Kit and the TaqMan miRNA Multiplex RT Assays, Human pool A, B (V2.1, V3.0, respectively). 3ul RNA was added to each reaction and RT-PCR was carried out on ABI Veriti Thermal cycler. After that we selected two ET and their corresponding NE preamplification products to perform preamplification and miRNA array analyses. 2.5ul of the product from the two paired samples was preamplified per manufacturer's protocol with the Megaplex PreAmp Primers (10×), Human pool A, B (V2.1, V3.0, respectively) and TaqMan PreAmp Master Mix (2×). In miRNA array, the expression was profiled with TaqMan Human microRNA arrays (V2.1 for pool A and V3.0 for pool B), using the manufacturer's recommended protocol (Applied Biosystems, Foster City, CA, USA).

The products from remaining 10 cases with EMPD, 10 cases of normal epidermis, and 7 cases of apocrine glands (the apocrine glands could not be found in every case) were used to perform real time-PCR. (TaqMan RT-PCR primers) to detect the specific miRNA expression levels according to the miRNA array data. Analyses of real time-PCR data were then conducted.

Data analysis

For miRNAs that were observed in all samples, cycle threshold (Ct) values were obtained with SDS 2.3 and RQ manager 1.2 software (Applied Biosystem), and ΔCt values were used in the statistical analysis. Differential expression of miRNAs were calculated with StatMiner® 4.2 (Integromics® Inc., Philadelphia, PA).

For the two pairs of samples in the array examination, all -ΔCt [$-(Ct-Ct_{RNU48})$] was calculated and heat map analysis was performed with hierarchical clustering.

The meanvalue of -ΔCt each miRNA in both groups (EMPD and normal epidermis) and ΔΔCt ($\Delta Ct_{ET} - \Delta Ct_{NE}$) were calculated. Those 4 sorts miRNAs that |ΔΔCt|>4 was also selected out. Histograph was performed by GraphPad Prism.

In conformation test for representive miRNAs together with determining apocrine glands expression of those miRNAs, using Bonferroni's Multiple Comparison Test to compare the (-ΔCt) value among the three groups. P-value < 0.05 are considered significant different between two groups. Scattergraph was performed by GraphPad Prism.

SUPPLEMENTARY FIGURE AND TABLE

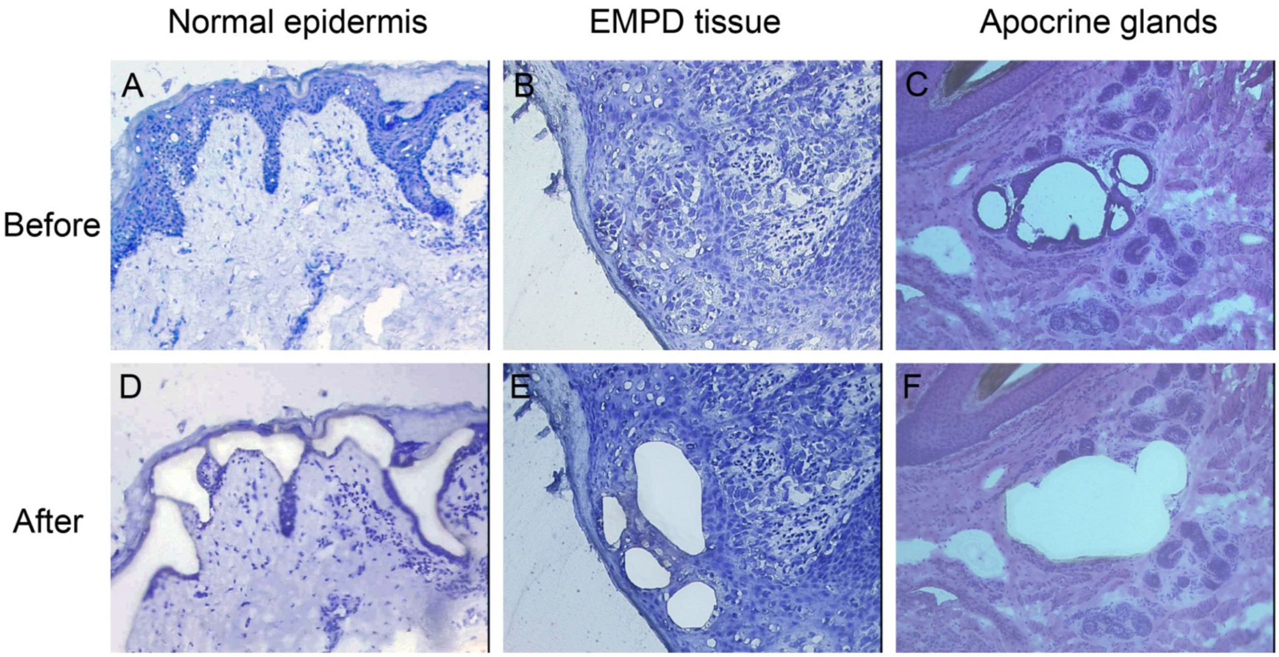

Supplementary Figure S1: Precise tissue selection via laser capture micro-dissection. A. Normal epidermis specimen before micro-dissection (*200 haematoxylin). **B.** EMPD tissue specimen before micro-dissection (*200 haematoxylin) **C.** Normal apocrine glands specimen before micro-dissection (*200 HE) **D.** Normal skin specimen after collection of epidermis via micro-dissection (*200 haematoxylin). **E.** EMPD tissue specimen after collection of Paget cells via micro-dissection (*200 haematoxylin). **F.** Normal apocrine glands specimen after collection of the apocrine glands via micro-dissection (*200 HE).

Supplementary Table S1: General information of selected EMPD patients.

See Supplementary File 1
