# Supplementary material for: miR-31 is distinctively overexpressed in primary male extramammary Paget's disease
Source: Oncotarget. 2016 Mar 21;7(17):24559–63. doi: 10.18632/oncotarget.8230 (PMC5029722; doi:10.18632/oncotarget.8230)
Supplement: Supplementary file 2 [file oncotarget-07-24559-s002.docx]

Supplementary Tab 1. General information of selected EMPD patients

| **No.** | **Gender** | **Age (years)** | **Tissue selection （via laser capture micro-dissection）** | **application** | **Duration (years)** | **Lesion location** | **Clinical manifestation** | **Distribution of paget's cells** |
| --- | --- | --- | --- | --- | --- | --- | --- | --- |
| 1 | male | 79 | EMPD tissue group and normal epidermis group | miRNA array | 5 | scrotum and inguinal region | an erythema with small amount of exudation about 10*7 cm in size in left gorin and scrotum, a nodule about 6*3cm presented on the center of the erythema | within epidermis |
| 2 | male | 79 | EMPD group , normal epidermis group and apocrine glands group | miRNA array | 1.5 | penis and scortum | an erythema with pus bloody exudation about 5*3 cm in size presented in the mons，penis and scortum area | within epidermis |
| 3 | male | 59 | EMPD group , normal epidermis group and apocrine glands group | real-time PCR | 8 | scortum | an erythema with mild erosion and small amount of exudation about 4*3 cm in size,a infiltrative plaque about 1*1cm presented on the erythem in scrotum | within epidermis |
| 4 | male | 62 | EMPD group , normal epidermis group and apocrine glands group | real-time PCR | 10 | scortum | an erythema with mild erosion and exudation about 4*6 cm in size,two round infiltrative plaques about 2*1.5cm presented on the erythema | within epidermis |
| 5 | male | 75 | EMPD group , normal epidermis group and apocrine glands group | real-time PCR | 0.5 | penis and scortum | skin hypertrophy of the scrotum and penis with several papules and plaques presented | papillaries involved |
| 6 | male | 70 | EMPD group and corresponding normal epidermis group | real-time PCR | 5 | scortum | an erythema about 10*8 cm in size with the edge dark red in color and small amount of exudation in the middle of the erythematous in scrotum | within epidermis |
| 7 | male | 66 | EMPD group and corresponding normal epidermis group | real-time PCR | 4 | scortum | an erythema with exudation about 5*3 cm in size in scrotum | within epidermis |
| 8 | male | 56 | EMPD group , normal epidermis group and apocrine glands group | real-time PCR | 3 | mons and scortum | a clear boundary erythema with mild erosion and exudation about 4*4 cm in size,a verrucous plaque about 1*1cm presented on the erythema in the mons and scrotum area | papillaries involved |
| 9 | male | 68 | EMPD group and corresponding normal epidermis group | real-time PCR | 3 | scortum | a dark red palque with rough surface without erosion about 1.5*1 cm in size | within epidermis |
| 10 | male | 71 | EMPD group and corresponding normal epidermis group | real-time PCR | 2 | scortum | a dark red infiltrive palque with clear boundary about 4*4 cm in size | papillaries and eccrine ducts involved |
| 11 | male | 60 | EMPD group , normal epidermis group and apocrine glands group | real-time PCR | 2 | scortum | an erythema with erosion and bloody exudation about 4*2.5 cm in size | within epidermis |
| 12 | male | 64 | EMPD group , normal epidermis group and apocrine glands group | real-time PCR | 12 | scortum | clear boundary erythematous with erosion and exudation about 4*2.5 cm in size | dermis and appendages involved |
